# A spacer infection by *Candida albicans* secondary to a *Staphylococcus capitis* prosthetic joint infection: a case report

**DOI:** 10.1186/s12879-021-06113-8

**Published:** 2021-05-04

**Authors:** Marta Bottagisio, Alessandro Bidossi, Nicola Logoluso, Antonio Pellegrini, Elena De Vecchi

**Affiliations:** 1 IRCCS Istituto Ortopedico Galeazzi, Laboratory of Clinical Chemistry and Microbiology, Via R. Galeazzi 4, 20161 Milan, Italy; 2 IRCCS Istituto Ortopedico Galeazzi, Chirurgia Ricostruttiva e delle Infezioni osteo-articolari (C.R.I.O), Via R. Galeazzi 4, 20161 Milan, Italy

**Keywords:** Case report, Spacer infection, *Candida albicans*, *Staphylococcus capitis*, Total hip arthroplasty, Prosthetic joint infection, Orthopedics

## Abstract

**Background:**

Prosthetic joint infection (PJI) is one of the most feared complications following total arthroplasty surgeries. Gram-positive bacteria are the most common microorganisms implicated in PJIs, while infections mediated by fungi only account for 1% of cases. When dealing with PJIs, a two-stage revision arthroplasty is widely used. Briefly, a spacer is introduced until re-implantation of the definitive prosthesis to provide skeleton stabilization while delivering antibiotics in the site of the infection. Sometimes, antimicrobial therapy may fail, but the isolation of a second microorganism from the spacer is uncommon and even less frequent that of a yeast.

**Case presentation:**

Here is described a case of a 75-year-old woman who underwent two-stage revision surgery of the left hip prosthesis secondary to a *Staphylococcus capitis* infection, whose spacer was found to be infected by *Candida albicans* at a later time. Briefly, the patient underwent revision surgery of the hip prosthesis for a suspected PJI. After the debridement of the infected tissue, an antibiotic-loaded spacer was implanted. The microbiological analysis of the periprosthetic tissues and the implant depicted a *S. capitis* infection that was treated according to the antimicrobial susceptibility profile of the clinical isolate. Three months later, the patient was admitted to the emergency room due to local inflammatory signs. Synovial fluid was sent to the laboratory for culture. No evidence of *S. capitis* was detected, however, a yeast was identified as *Candida albicans*. Fifteen days later, the patient was hospitalized for the removal of the infected spacer. Microbiological cultures confirmed the results of the synovial fluid analysis. According to the susceptibility profile, the patient was treated with fluconazole (400 mg/day) for 6 months. Seven months later, the patient underwent second-stage surgery. The microbiological tests on the spacer were all negative. After 12 months of follow-up, the patient has fully recovered and no radiological signs of infection have been detected.

**Conclusions:**

Given the exceptionality of this complication, it is important to report these events to better understand the clinical outcomes after the selected therapeutic options to prevent and forestall the development of either bacterial or fungal spacer infections.

## Background

Prosthetic joint infection (PJI) is one of the most feared complications following total arthroplasty surgeries. The presence of a foreign body is the triggering event for infections, because it permits the attachment of microorganisms and biofilm formation, making the eradication of the infection difficult [1]. Gram-positive bacteria are the most common microorganisms implicated in PJIs, while infections mediated by fungi only account for 1% of cases in which *Candida albicans* is the leading organism [2]. When dealing with PJIs, a two-stage revision arthroplasty is widely used. Although being an invasive treatment, two-stage revision arthroplasty has a rate of success that ranges from 70 to 95% [3]. The two-stage procedure involves the introduction of a spacer until the re-implantation of the definitive prosthesis to provide skeleton stabilization while delivering a massive amount of antibiotics in the site of the infection. However, in some cases, antimicrobial therapy may fail to eradicate the pathogen causing the persistence of infection [4, 5]. Uncommon is the isolation of a second microorganism from the spacer and even less frequent that of a yeast. Here is described the case of a patient who underwent two-stage revision surgery of the left hip prosthesis secondary to a *Staphylococcus capitis* infection, whose spacer was found to be infected by *Candida albicans* at a later time.

## Case presentation

A 75-year-old female underwent left total hip arthroplasty secondary to severe osteoarthritis in 1989 with a subsequent revision surgery due to pain. The patient has a history of hypercholesterolemia and no predisposing comorbidities or risk factors (e.g. rheumatoid arthritis, immune-suppressive therapy with steroids, liver cirrhosis, etc.).

**Fig. 1:**
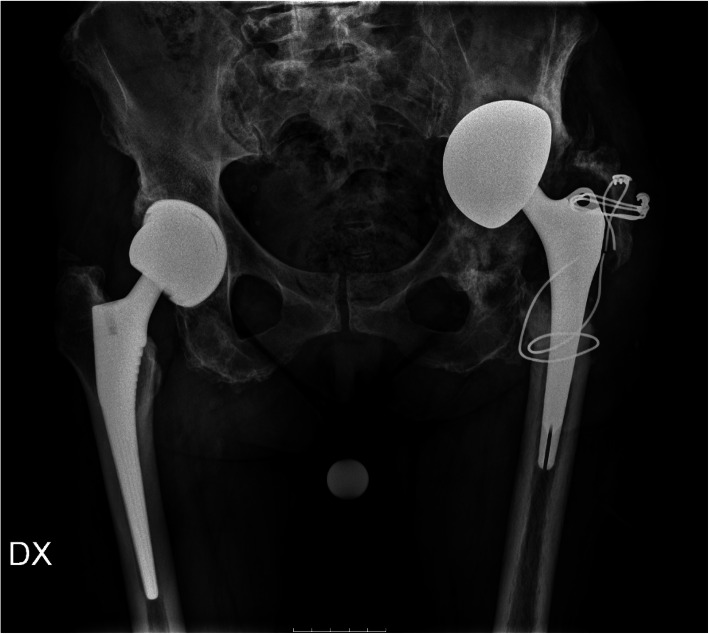
Radiological signs of bone loosening before revision surgery of the left hip prosthesis

In November 2018, the patient was admitted to the hospital due to the appearance of secreting sinus, subsequently treated by antibiotic therapy without any improvement. Physical examinations revealed the presence of inflammatory signs (i.e. heat, pain, and redness). Radiographs highlighted bone loosening with the migration of prosthetic components (Fig. 1). Laboratory tests showed a C-reactive protein (CRP) of 4.43 mg/l and an erythrocyte sedimentation rate of 88 mm/hour.

The next day, the patient underwent revision surgery of the left hip prosthesis for a suspected PJI. After the debridement of the infected soft tissue, a Vancogenx-Space (Tecres S.p.a.) spacer loaded with vancomycin and gentamicin was fixed with Simplex™ bone cement (Stryker) with erythromycin and colistin, further enriched with 3 g vancomycin. Vancomycin (1 g/2 days) and meropenem (1 g/2 days) was used in peri-operative prophylaxis. Histological analysis depicted a chronic inflammation with fibroconnective tissue speculating an ongoing metallosis. Three periprosthetic tissue samples and the implant itself were collected intraoperatively and sent to the laboratory for culture. Aliquots of eluate obtained after treatment with 0.1% dithiothreitol (DTT, Sigma-Aldrich) were then cultured in proper conditions [6]. After incubation, all the samples yielded the growth of Gram-positive cocci identified as *Staphylococcus capitis* by means of Vitek2 System (BioMeriéux). The antibiotic treatment started the day of surgery with intravenous antibiotics (vancomycin 1 g every 12 h and meropenem 1 g every 8 h) and shifted to vancomycin alone after the antimicrobial susceptibility profile was obtained. It was suspended due to cutaneous erythema 10 days after. Therefore, based on antimicrobial susceptibility results, treatment was continued with oral trimethoprim/sulfamethoxazole (160/800 mg every 12 h for 6 weeks). During the hospitalization period, urinalysis and urine culture were routinely performed without evidencing any sign of infection due to bacteria or yeasts. Nineteen days after surgery CRP levels decreased and the patient was discharged from the hospital.

However, 3 months later, the patient was attended to the emergency room due to the presence of inflammatory signs (i.e. edema and swelling). She did not report any infectious event in the period from hospital discharge to the admission to emergency department when specifically asked. Synovial fluid was collected and sent to the laboratory for culture. The sample was plated on agar plates and inoculated into proper broths. No evidence of *S. capitis* was detected, however, a yeast grew on Chocolate agar and Sabouraud agar plates and after 3 days, also in BHI broth. The yeast was identified as *Candida albicans* by Vitek2 system. The retrieved *C. albicans* was susceptible to all the tested antifungal agents (amphotericin B MIC = 0.5 mg/l; caspofungin MIC ≤0.12 mg/l; flucytosine MIC ≤0.1 mg/l; fluconazole MIC ≤0.5 mg/l; micafungin MIC ≤0.06 mg/l; voriconazole MIC ≤0.12 mg/l).

Fifteen days later, the patient was hospitalized for the removal of the infected spacer. Briefly, a custom-made spacer was created with StageOne™ Select Hip Cement Spacer Molds (Zimmer Biomet) using the Refobacin® Bone Cement R (Zimmer Biomet) containing gentamicin and clindamycin and further enriched with 400 mg voriconazole. Afterward, the explanted spacer was sent to the laboratory together with biopsies of the surrounding soft tissues and the synovial fluid. The synovial fluid analysis evidenced a leukocyte count of 2926 cells/μl with 74.3% of polymorphonuclear cells. Leukocyte esterase resulted positive (score 2+). Microbiological cultures confirmed the results of the synovial fluid sample collected in the emergency room with the isolation of *C. albicans* with the same susceptibility profile. Again, cultures were negative for *S. capitis*. According to the susceptibility profile, the patient was treated with fluconazole (400 mg/day). Additionally, 1 g vancomycin and 1 g meropenem until the results of the intra-operative cultural examination were administered. The same therapeutic plan was followed once the patient was discharged from the hospital for the next 6 months. In November 2019, the patient underwent second-stage surgery, performed with an un-cemented hip revision implant (multihole acetabular component also fixed with screws and diaphyseal stem with distal femoral grip) to overcome the huge loss of bone substance as a consequence of the infection and previous interventions. The removed spacer was sent to the laboratory and the microbiological tests were all negative, hence, after 15 days the antifungal and antibiotic therapy was suspended. After 12 months of follow-up, the patient has fully recovered and no radiological signs of infection have been detected.

## Discussion and conclusions

The most frequently encountered bacteria isolated from PJIs undoubtedly belong to Staphylococcus spp. genus, of which coagulase-negative staphylococci (CoNS) occur in 13–41% of PJIs [7]. Although *Staphylococcus epidermidis* and *Staphylococcus lugdunensis* are the most documented species, PJIs mediated by *Staphylococcus capitis* have been described in the literature [7]. Suffice it to say that the number of cases mediated by *S. capitis* diagnosed from January 2013 to June 2015 in our Orthopedic Institute alone was 33 (17 hip and 16 knee infections), accounting for the 6.5% of all the PJI cases recorded [8].

Despite rare, fungal infections are one of the most feared complications following the implant of an orthopedic device. The majority of PJIs mediated by yeasts is caused by *C. albicans* due to biofilm formation upon the surface of medical devices and possibly due to the composition of the matrix which differs from that produced by other *Candida* species (i.e. *C. parapsilosis*, *C. tropicalis*, and *C. glabrata*) [9]. As reported in the literature, the incidence of *C. albicans*-mediated PJIs is around 1% [2].

Differently from bacterial-mediated infections, fungal PJIs are usually associated with mild clinical signs, thus the diagnosis can be often delayed [10]. As observed in our clinical case, lower CRP levels were detected at the time of the second hospitalization for the removal of the infected spacer compared to the first PJI mediated by *S. capitis* (2.19 mg/l and 4.43 mg/l, respectively).

The sooner the cause of PJI is recognized, the better the outcome will be because the time of diagnosis plays a crucial role in determining the fate of the two-stage procedure, recommended in the case of an infection mediated by fungi [11]. In this context, it is mandatory to consider additional diagnostic tools and procedures to analyze the collected tissue samples. It is recommended the use of protocols able to support the growth of the most common yeast [12]. Especially when antibiotic-loaded spacers are implanted, it must be paid extra attention to all the microorganisms not affected by the antibiotic used. Even if the amount of local antibiotics released from spacers is higher compared to that detected after systemic administration of drugs, the medication is effective only if the pathogens are susceptible to the loaded molecules [13]. Not to mention, that spacers are usually loaded with antibacterial compounds rather than antifungals. Indeed, the physical characteristics of the spacers and the inflammatory environment caused by the chronic infection and surgery, together with the local antibiotic therapy unsuited to forestall fungal colonization might have predisposed and supported the infection by *C. albicans* reported in the present clinical case report. Such factors might have triggered the switch from yeast to hyphal form, necessary for the transition to a sessile state [14]. Clinical cases reporting the bacterial colonization of spacers following a two-stage arthroplasty are scarcely reported in the literature [15, 16]. To the best of our knowledge, no studies reporting the presence of yeasts on a spacer implanted following a two-stage revision surgery exist.

Compared to bacterial infection, the success rate of fungal PJI is lower and longer antifungal treatments are required, increasing the risk of development of resistance [17]. The local use of liposomal amphotericin B or azole can be effective and relatively safe for prolonged administration to treat Candida-mediated PJIs. However, voriconazole is not the best-recommended option because it confers a loss of mechanical strength when fabricating spacers [12]. The combination of antifungal and antibacterial agents might be a stronger precautionary approach when dealing with patients with comorbidities or factors predisposing to the development of fungal PJIs. Finally, given the uncommonness of these complications, it is important to report these cases to better understand the clinical outcomes after the selected therapeutic option.

## Data Availability

The data that support the findings of this study are available on request from the corresponding author. The data are not publicly available due to privacy or ethical restrictions.

## Abbreviations

PJI: Prosthetic Joint Infections
CRP: C - reactive protein
BHI: Brain Heart Infusion
MIC: Minimum Inhibitory Concentration
